# Precision Proteolysis of Triosephosphate Isomerase of *Escherichia coli* Boosts Dihydroxyacetone Phosphate Biosynthesis

**DOI:** 10.1021/acssynbio.5c00870

**Published:** 2026-03-03

**Authors:** Belén Calles, Daniel C. Volke, Max Chavarría, Pablo I. Nikel, Víctor de Lorenzo

**Affiliations:** † Systems Biology Department, Centro Nacional de Biotecnología-CSIC, Campus de Cantoblanco, Madrid 28049 Spain; ‡ The Novo Nordisk Foundation Center for Biosustainability, 5205 Technical University of Denmark, Kongens Lyngby 2800, Denmark; § Escuela de Química and CIPRONA, Universidad de Costa Rica, San José 2060 Costa Rica

**Keywords:** proteolysis, triosephosphate isomerase, DHAP, NIa protease, glycolysis

## Abstract

Dihydroxyacetone phosphate (DHAP), a key metabolic intermediate of the Embden–Meyerhof–Parnas pathway of *Escherichia coli*, has a considerable value as a precursor of high-added-value compounds. While eliminating the triosephosphate isomerase (*tpiA*) gene should theoretically channel 50% of the glycolytic flux into dead-end production of DHAP, the permanent loss of this activity triggers alternative routes that decrease (rather than increase) DHAP levels. To address this limitation and establish transient regimes of high DHAP biosynthesis, we harnessed the unusual structural tolerance of TpiA for designing a variant of the enzyme that can be rapidly degraded, thus temporarily adopting a null phenotype. This was achieved through conditional expression of the highly specific viral protease PPV-NIa, which cleaves a cognate recognition sequence strategically engineered into an exposed, permissive loop on the protein surface. Optimization of such an *in vivo* proteolytic device resulted in fully active TpiA variants that become nearly instantly destroyed upon induction of NIa *in trans*, which was itself engineered as an ON/OFF switch. Metabolomic data of an engineered *E. coli* strain genomically encoding the cognate genetic device showed that precise post-transcriptional targeting of TpiA leads to a substantial transitory increase of DHAP with minimal disturbance of other typical intermediates. The general value of targeting enzymes in central carbon metabolism, such as TpiA, is discussed in light of systems metabolic engineering.

## Graphical abstract

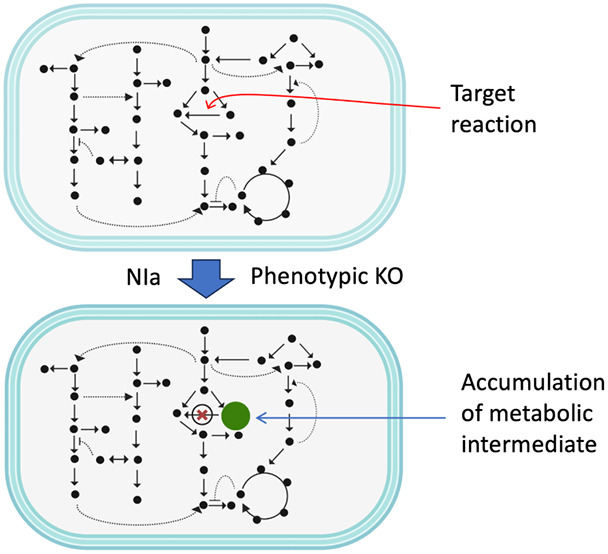

Bacterial metabolism is a rich source of molecules of considerable interest either by themselves or as precursors of chemical compounds of high-added value. One such molecule is dihydroxyacetone phosphate (DHAP), one of the two products of the breakdown of fructose 1,6-bisphosphate, along with glyceraldehyde-3-phosphate (GAP) through the canonical Embden–Meyerhof–Parnas (EMP) pathway (Figure 1). From a biotechnological perspective, DHAP-dependent aldolase-catalyzed reactions^1^ can be efficiently coupled in multistep processes to enable the asymmetric synthesis of carbohydrates, iminosugars, cyclitols, and a broad range of complex natural and synthetic products (reviewed in refs 2–5 ).

**Figure 1:**
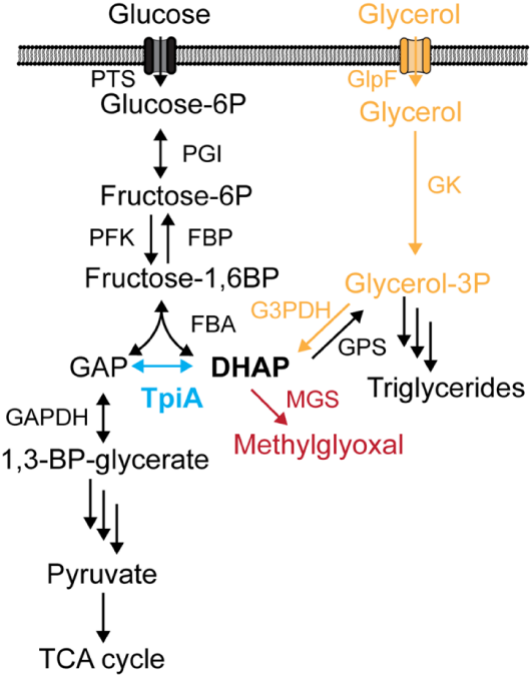
Metabolic context of DHAP production. Blowup of the metabolic node for DHAP generation and its connections. The most prominent enzyme hampering dihydroxyacetone phosphate (DHAP) accumulation is triosephosphate isomerase (TpiA). TpiA normally converts DHAP into glyceraldehyde-3-phosphate (GAP), which is crucial for the downstream steps of both glycolysis and the conversion of glycerol to energy. Without TpiA, the conversion of DHAP to GAP is blocked, effectively creating a bottleneck in the metabolic pathway, preventing the efficient flow of carbon necessary for growth on glycerol. The methylglyoxal pathway can be activated to prevent accumulation of DHAP under conditions of metabolic imbalance. GAP, glyceraldehyde-3-phosphate; DHAP, dihydroxyacetone phosphate; PTS, phosphotransferase system; PGI, glucose-6-phosphate isomerase; PFK, phosphofructokinase; FBP, fructose bisphosphatase; FBA, fructose bisphosphate aldolase; TpiA, triosephosphate isomerase; GAPDH, glyceraldehyde-3-phosphate dehydrogenase; MGS, methylglyoxal synthase; GPS, glycerol-3-phosphate synthase; G3PDH, glycerol-3-phosphate dehydrogenase; GK, glycerol kinase; GlpF, glycerol uptake facilitator.

Despite interest in this building block for complex chemicals, DHAP production remains a substantial challenge. Steady-state DHAP intracellular concentrations are kept low because it is rapidly and reversibly isomerized to GAP, which follows down the catabolic EMP route (Figure 1). Current methods for the generation of GAP and DHAP are expensive and inefficient. Chemical synthesis, for instance, involves complex multistep procedures, including protection and deprotection steps, and often requires the use of hazardous reagents, leading to low yields and significant environmental impact. In this context, biological approaches to DHAP synthesis are particularly appealing due to their efficiency, with many offering one-pot processes and some even enabling one-step routes to the phosphorylated product.^6^ Ideally, DHAP could be produced *in vivo* and benefit from advanced large-culture growth and extraction available for bacterial platforms.^7–9^ Yet, this is not straightforward because, as indicated above, all DHAP produced from glucose in *E. coli* is converted into GAP through the action of the triosephosphate isomerase (TpiA) enzyme,^10,11^ so there is virtually none of the compound to be recovered from wild-type cells. Furthermore, while deletion of *tpiA* creates a dead-end in the glycolytic pathway that should lead to DHAP accumulation, such a rise in the concentration is transient due the activation of methylglyoxal synthase (MgsA) that converts the triose to pyruvate^12^ (Figure 1). As a matter of fact, the glycolytic flux is split between lower glycolysis and the methylglyoxal branch in a *tpiA-*defective strain when cells grow in glucose.^13–16^ MgsA requires homotropic activation by elevated DHAP concentrations and is strongly inhibited by free phosphate.^17^ Hence, during homeostatic growth, methylglyoxal formation is maintained at low levels, and the slow activation of MgsA offers a window of opportunity for DHAP accumulation provided that the TpiA activity is quickly inactivated. Furthermore, a *tpiA* knockout strain has serious growth defects, making it problematic to use this mutant as a platform for DHAP production. Against this background, we entertained the idea that specifically and promptly inhibiting TpiA in an otherwise wild-type *E. coli* background could peak DHAP accumulation without affecting the remainder of the cell biochemistry.

Plenty of contemporary genetic tools have been developed in the past decade to cause conditional expression of genes of interest in bacteria, including inducible/repressible promoters,^18^ optogenetic devices,^19^ antisense, translation-inhibiting sRNAs,^20^ and dCas9-mediated interference and related CRISPR technologies.^21,22^ Yet, most of these systems take time between the exposure of cells to the inducing signal and the manifestation of the desired phenotype, and their implementation usually comes at the price of metabolic burden and/or toxicity issues. What could then be a feasible approach for the fast removal of TpiA for the sake of increasing intracellular DHAP accumulation?

Here, we present a proof-of-concept strategy that combines previously identified permissive insertion sites in TpiA^2,3^ with a tightly regulated genetic switch^2,4^ to enable conditional, protease-mediated elimination of an engineered enzyme in vivo. This design affords rapid and precise post-translational control of TpiA abundance, resulting in precise temporal control of DHAP buildup with minimal disturbance of the rest of the metabolome.

## Results and Discussion

### Rationale of Targeted Hit-And-Run Proteolysis for Controlling TpiA Activity *In Vivo*

The core principle of the rapid, post-translational enzyme inactivation strategy we term *hit-and-run proteolysis* is outlined in Figure 2. The approach relies on identifying permissive positions in the enzyme’s three-dimensional structure where a cleavage site for a highly specific protease can be introduced. This is achieved by genetically inserting the short sequence recognized by the protease into the corresponding coding sequence of the target gene. Importantly, this modification leaves the enzyme’s catalytic activity unaltered as long as the protease is absent from the intracellular environment. Thus, under normal conditions, no metabolic or phenotypic changes are observed. To implement conditional control, the protease is expressed from a tightly controlled inducible system. In the absence of the chemical inducer, no protease is produced, and the engineered enzyme remains fully active. Under these conditions, host cells are phenotypically indistinguishable from the wild-type. Upon induction, the thereby expressed protease cleaves off the engineered site in the enzyme, irreversibly abolishing its activity. This generates a temporary knockout phenotype that lasts as long as the protease is expressed.

**Figure 2:**
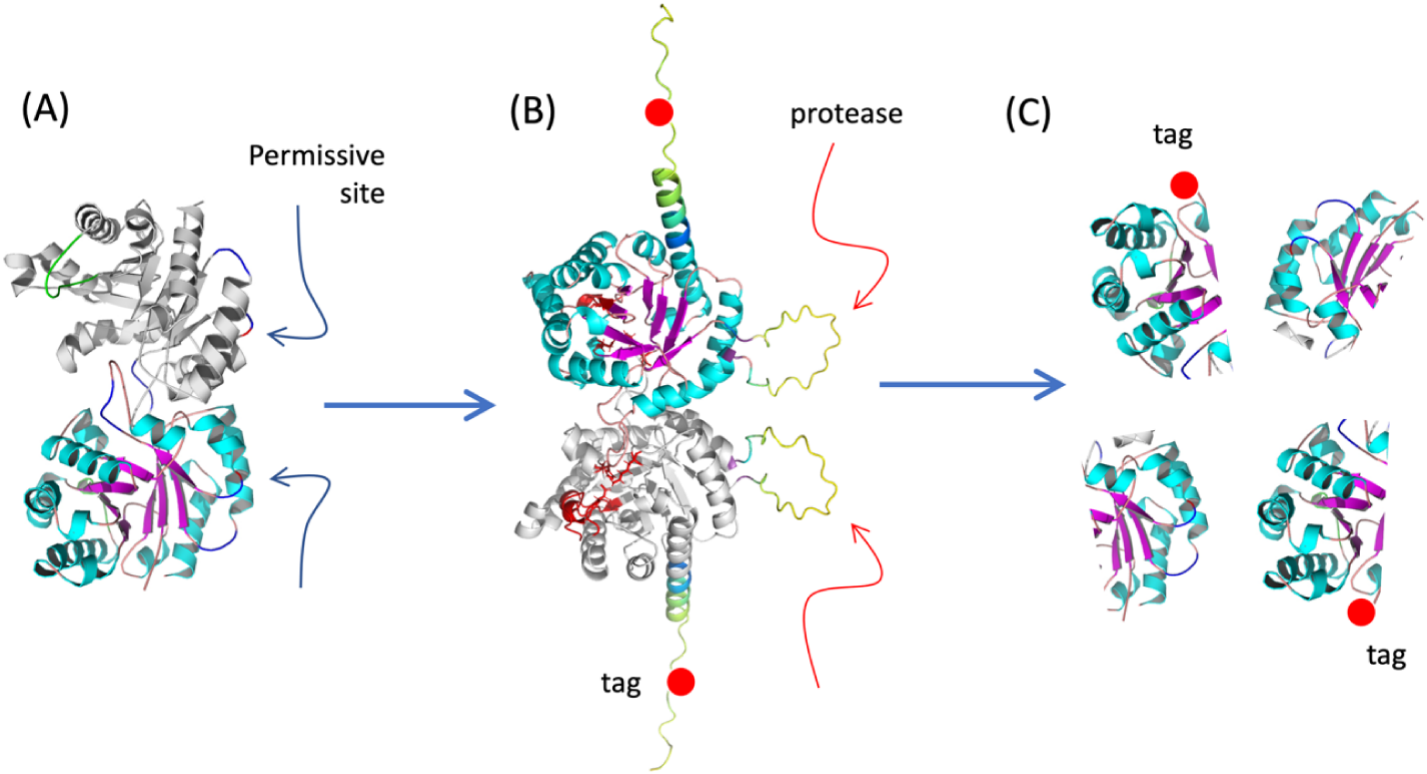
Hit-and-run proteolysis strategy for phenotypic knockout of target enzymes. (A) The approach begins by identifying permissive sites in the protein of interest (in our case TpiA) either by structural inspection or by scanning mutagenesis through the cognate gene sequence.^24^ (B) One of these permissive locations is then used to genetically anchor a peptide that is presented on the protein surface and acts as a recognition site for a highly specific protease, for example, NIa. For the purpose of monitoring *in vivo* expression, the protein can also be decorated with an immuno-tag (shown as a red dot) that facilitates detection and visualization of its integrity. (C) Conditional expression of the protease *in vivo* causes splitting of the protein into inactive fragments, whose occurrence can be detected via Western blot assays. Cleavage of the protein also entails a virtually instant loss of enzymatic activity.

As the objective of this work was to establish transient DHAP accumulation, TpiA is the enzyme of interest. As indicated above, DHAP can be produced stoichiometrically from glucose via glycolysis in a metabolic setup where TpiA is inactive if the metabolite is not processed by MgsA to yield methylglyoxal, as proven in a cell-free system.^25^ *In vivo*, GAP generated stoichiometrically from glucose alongside DHAP is converted to pyruvate and then to lactate. This sequence of reactions is expected to sustain an overall energy balance. In order to implement the conditional proteolysis system proposed above, the strategy involves endowing a permissive site of the TpiA structure with a recognition site of NIa, a highly specific protease involved in processing the potyvirus polyprotein.^26,27^ The phenotypic knockout of such a proteolizable TpiA variant is then brought about by the induction of the corresponding protease that cleaves the enzyme at the cognate sequence. The core issue is therefore the engineering of a fully active TpiA that can be also fully and specifically eliminated *in vivo* through targeted proteolysis with NIa.

### Probing Availability of Permissive Sites of Triosephosphate Isomerase to Proteolytic Cleavage

To endow TpiA with a functional NIa recognition site, we leveraged our previous study of structural permissiveness of the enzyme in which the whole primary sequence was scanned *in vivo* with pentapeptide insertions (15-bp), followed by testing functional activity *in vivo*.^23^ On this basis, we considered three parameters for optimal structural grafting of the NIa-cleavage sequence: [i] optimal insertion site, [ii] length of the peptide recognized by the protease, and [iii] extent of presentation of the target sequence on the protein surface. Although many known insertion-tolerant sites are mapped—most of them in otherwise structured regions—we sampled the ones that looked more promising for our purpose. We reasoned that larger insertions (e.g., the NIa cleaving site, see below) would be more likely to remain nondisruptive in a location near one of the naturally occurring loops, e.g., the one named L2, which tolerates insertions after the E55 residue^23^ (Figure 3). The quaternary structure of TpiA^28^ (PDB file 1TRE) shows that this location is far from the catalytic center of the enzyme (E167-A178 residues of the primary sequence).^29–31^ In addition, the TpiA region by the E55 residue is solvent-exposed^23^ and thus potentially available to the action of the protease. Finally, E55 is about halfway of the protein sequence, and splitting at that site could likely eliminate its activity altogether. Taking all of these considerations together, we first focused on the protein site around residue E55 as the preferred location to introduce the NIa target sequence.

**Figure 3:**
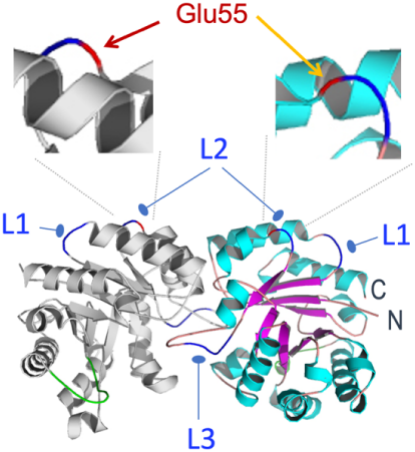
3D architecture of the triosephosphate isomerase protein dimer, showing the typical 8-fold (βα)-TIM barrel structure. Exposed loops (colored in dark blue) can be targeted for insertion of the protease recognition peptide. The L3 loop is too close to the active center of the enzyme (colored in green) and to the dimer interface. The zoom-in above shows the permissive insertion site located in L2 at position E55 (colored red).^23^

To introduce the NIa site within the selected position, we made use of the unique *Pme*I site included in the 15-bp insertion resulting from the earlier linker scanning mutagenesis (TpiA variant E55; see Materials and Methods). The final insert encoded a 13 amino acid long peptide GCL·**NVVVHQA**·KNK, where the central heptapeptide NVVVHQA is the conserved PPV NI_b_-CP (NIa F) recognition site and the flanking sequences were carried on from the earlier pentapeptide insertion^32^ (Table S2). Besides the peptide insertion, the engineered TpiA (as well as all other variants described below) was translationally fused to a C-terminal E-tag for monitoring expression *in vivo*.^23^ The resulting protein variant was called TpiA^E55^·^1^, and its functionality was tested first *in vivo* with a simple complementation assay. An *E. coli* strain defective in *tpiA* (*E. coli* W3110 Δ*tpiA*) was, as expected, unable to grow in minimal medium supplemented with glycerol as the sole carbon source (Figure 4A). Yet, growth was fully restored to wild-type levels when TpiA^E55·1^ was produced *in trans* from an expression plasmid (pBCL3-E55·1), indicating that the NIa insertion did not affect its catalytic performance. In addition to this *in vivo* test, the *in vitro* enzymatic activity of TpiA^E55·1^ was verified in soluble extracts obtained both from the null and the complemented strains, using lysates of the parental strain *E. coli* W3110 as a reference. As expected, the wild-type and the complemented strains displayed comparable levels of TpiA activity, whereas the knockout strain did not (Figure 4B). Note also that TpiA activity was higher in the strain carrying the *tpiA* gene in plasmids, plausibly reflecting the increased amounts of enzyme in each of these protein extracts. These results confirmed that NIa site insertion at position E55 of the TpiA protein had no effect on its activity. Next, we tested the amenability of the TpiA^E55·1^ variant to *in vivo* proteolysis by NIa by cotransforming the Δ*tpiA* strain with plasmid pBCL3-E55·1 (Table 1) and a compatible construct (pPPV1) that expresses the NIa protease under the control of an IPTG-inducible promoter (Figure 4C). Alas, cleavage of the TpiA^E55·1^ variant in this configuration was negligible. In contrast, a control experiment run with a fragment of the plum pox virus polyprotein containing the natural NIa target site (encoded by plasmid pPPV30 ^33^) was efficiently cleaved under the same conditions, demonstrating that the NIa protease expressed from pPPV1 *in vivo* was indeed active (Figure S1).

**Figure 4:**
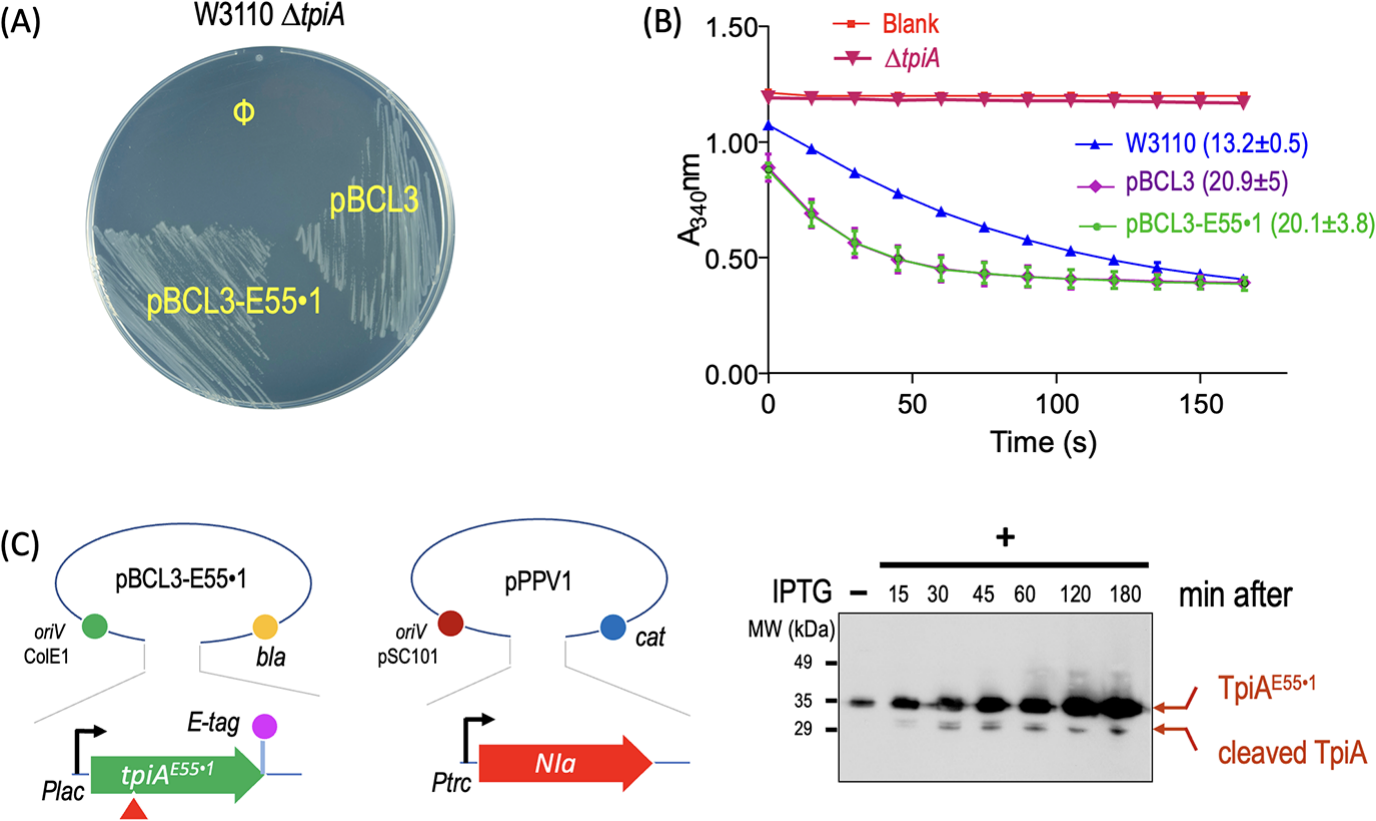
Functional complementation and activity analysis of TpiA^E55·1^. (A) The *E. coli* W3110 Δ*tpiA* strain was transformed with plasmid pUC18Not/T7* carrying either the TpiA-Etag wild-type-like variant (TpiA^ET^, encoded in plasmid pBCL3) or the same construct containing the NIa seven-amino-acid target sequence inserted into the pentapeptide loop located after residue E55 (pBCL3-E55·1). The empty plasmid pUC18Not/T7* (Φ) served as the negative control. Growth complementation was assessed on minimal medium plates containing 1% glycerol as the sole carbon source. (B) Enzymatic activity of the strains described in panel A, along with the *E. coli* W3110 wild-type strain carrying the native chromosomal copy of *tpiA*. Two independent enzymatic assays (three replicas of each sample) were carried out as explained in the experimental procedures. Briefly, triosephosphate isomerase activity was monitored by using a coupled assay in which GAP was converted to DHAP that in turn produced glyerol-3-phosphate by the action of GDH, in a redox reaction that requires NADH whose consumption was measured by the decrease of absorbance at 340 nm. The slope of the curve directly correlates with TpiA activity. (C) Diagram of the two-plasmid expression system (left panel) employed to assess the performance of the TpiA^E55·1^ variant in response to NIa protease, expressed *in trans* from plasmid pPPV1, shown in the Western blot in the right panel. Expression from both plasmids was simultaneously induced with IPTG.

**Table 1:** Strains and Plasmids Used in This Work

Strain/plasmid	Relevant features	Reference
*E. coli*		
W3110	K12 F^–^ IN (*rrnD-rrnE*)	Lab collection
W3110Δ*tpiA*	*tpiA* deletion derivative of W3110 strain	^23^
XL1-Blue	*F′[proAB lacI* ^*q*^ *lacZΔM15 Tn10] endA1 hsdR17 supE44 thi-1 recA1 gyrA96 relA1, lac*	^62^
DH5α	F-, *supE44, ΔlacU169, (ϕ80 lacZDM15), hsdR17, (rk-mk+), recA1, endA1, thi1, gyrA, relA*	Lab collection
CC118λ*pir*	Δ(*ara*-*leu*), *araD*, Δ*lac*X174, *galE*, *galK, phoA, thi1, rpsE, rpoB, argE (Am), recA1,* lysogenic λ*pir*	^53^
BCL4	W3110 strain with the *tpi*A gene tagged with a C-terminal Etag epitope and inserted with a NIa site after E55 residue	This work
BCL3	W3110 strain harboring the *tpi*A gene tagged with a C-terminal Etag epitope (used as TpiA wt-like control)	This work
*Plasmids*		
pBCL3	pUC18Not/T7* based vector harboring TpiA^ET^, a *tpi*A gene with a C-terminal Etag fusion	^23^
pBCL3-E55	pBCL3 based plasmid harboring a pentapeptide insertion after E55 residue in tpiA gene	^23^
pBCL3-E160	pBCL3 based plasmid harboring a pentapeptide insertion after E160 residue in *tpiA* gene	^23^
pBCL3-A195	pBCL3 based plasmid harboring a pentapeptide insertion after A195 residue in *tpiA* gene	^23^
pBCL3-E55·1	pBCL3 based plasmid harboring a 13 aa long insertion, including NIa core site, after E55 residue in *tpiA* gene	This work
pBCL3-E160·1	pBCL3 based plasmid harboring a 13 aa long insertion, including NIa core site, after E160 residue in *tpiA* gene	This work
pBCL3-A195·1	pBCL3 based plasmid harboring a 12 aa long insertion, including NIa core site, after E55 residue in *tpiA* gene	This work
pBCL3-E55·NE	pBCL3 based plasmid, harboring a 23-amino-acid insertion composed of the NIa core cleavage site plus five flanking residues in their native arrangement, inserted after residue E55 in *tpiA* gene	This work
pBCL3-E55·FL	pBCL3-based plasmid harboring a 23-amino-acid insertion consisting of the NIa core site flanked by five Gly/Ser residues on each side, positioned after residue E55 in the *tpiA* gene.	This work
pBCL3-E55·NEΔ1	pBCL3 based plasmid, harboring a 21-amino-acid insertion composed of the NIa core cleavage site plus four flanking residues in their native arrangement, inserted after residue E55 in *tpiA* gene	This work
pBCL3-E55·NEΔ2	pBCL3 based plasmid, harboring a 19-amino-acid insertion composed of the NIa core cleavage site plus three flanking residues in their native arrangement, inserted after residue E55 in *tpiA* gene	This work
pBCL3-E55·NEΔ3	pBCL3 based plasmid, harboring a 17-amino-acid insertion composed of the NIa core cleavage site plus two flanking residues in their native arrangement, inserted after residue E55 in *tpiA* gene	This work
pBCL3-E55·NEΔ4	pBCL3 based plasmid, harboring a 15-amino-acid insertion composed of the NIa core cleavage site plus one flanking residues in their native arrangement, inserted after residue E55 in *tpiA* gene	This work
pBCL3-E55·NEΔ2–1	pBCL3 based plasmid, harboring a 18-amino-acid insertion composed of two flanking residues before and three after the NIa core cleavage site, in their native arrangement, inserted after residue E55 in *tpiA* gene	This work
pBCL3-E55·NEΔ2–2	pBCL3 based plasmid, harboring a 18-amino-acid insertion composed of three flanking residues before and two after the NIa core cleavage site, in their native arrangement, inserted after residue E55 in *tpiA* gene	This work
pKNG101	Sm^R^, *sacB* ^+^, *oriV* R6K, *oriT*, suicide delivery vector	^54^
pKNG101-tpiA	pKNG101 based vector harboring the *tpiA* ^*E55·NEΔ2*^ gene plus a 0.5 Kb downstream DNA fragment flanked by a I-SceI endonuclease site	^23^
pACBSR	*Cm* ^R^, p15A ori, araC, P BAD, I-SceI and λ Red genes	^55^
pPPV1	pVTR-B plasmid containing 0.6 Kb StuI-*Hin*dIII fragment encoding PPV NIa protease from pPPVs20 plasmid	^42^
pPPV30	pSU8 derivative containing the *Sal*I-*Pst*I fragment of PPV cDNA consisting of the 3′ terminal region from nt 3627	^33^
pS238D·NIa	Derivative of pSEVA238 harboring the potyvirus *nIa* protease gene under the control of the LacI-dependent digitalizing module	^24^

Given the artificial context of the NIa recognition sequence,^33,34^ this result was not altogether unexpected, but it prompted us to construct two additional TpiA variants bearing the NIa recognition site at different permissive sites of the protein structure, i.e., positions E160 and A195, which appeared often in the earlier linker scanning approach,^23^ rendering these variants strong candidates for effective surface display of the NIa sequence. As before, we also took advantage of the *Pme*I site left behind in these locations following the prior pentapeptide insertions (Materials and Methods and Table S2). The resulting plasmids (Table 1) pBCL3-E160·1 (encoding TpiA^E160·1^) and pBCL3-A195·1 (encoding TpiA^A195·1^) turned out to display wild-type-like isomerase activity *in vivo* (see complementation assays in Figure S2A) and *in vitro* (enzymatic activity; Figure S2B), indicating that TpiA folded properly despite being inserted with a 13-amino acid peptide within structured regions. The corresponding expression plasmids were introduced as before into the *ΔtpiA* strain along with plasmid pPPV1 and the extracts probed with an anti-E-Tag antibody. Unfortunately, none of them appeared to be amenable to proteolytic cleavage by NIa *in vivo* (Figure S2C). Such a lack of cleavage in three otherwise apparently optimal positions led us to hypothesize that the pentapeptide that acts as the substrate of the protease could not be protruding enough to become available for proteolytic action. A structural simulation of the access to the PPV-NIa protease of such a heptapeptide in the engineered TpiA context exposed that, indeed, the loop containing the insertion was too short (not shown), suggesting that an optimal positioning of the target site in the active center of the protease would require at least 7-amino acid extension of the tag flanked by at least four amino acids on both sides. But what sequences could work best for such lateral additions? While the NVVVHQA peptide seems to be the core target split by NIa, flanking sequences in the original context of the viral polyprotein seem to help efficient recognition and cleavage.^35,36^ Inspection of the amino acids at either side of the conserved heptapeptides constituting the NIa-cleaving sites A to F in potyvirus revealed high proportion of charged residues,^35,37^ which could provide an improved molecular context for NIa recognition. These considerations boil down to re-engineering the NIa sites in TpiA in terms of [i] modifying the protrusion of the protease-targeting peptide with respect to the rest of the protein body and [ii] amending the flanking amino acids to make the extension more polar. To this end, we focused on the E55 location of the TpiA protein that—as discussed above—was expected to be more permissive to such modifications.

### Optimization of a NIa Protease Cleavage Site in TpiA

The potential roles of enlarging the presentation of the protease target size on the TpiA surface and rearranging the polarity of the flanking sequences were addressed separately. This was enabled by the *Pme*I site mentioned available in TpiA^E55^ at residue E55. In one case, the NIa cleavage site was extended by adding GS linkers at either side of the conserved heptapeptide, i.e., the inset in TpiA^E55^ was GSGSG·**NVVVHQA·**GSGSG. Such repeats of glycine and serine residues were chosen because they form random coils, which do not interfere with the folding of adjacent protein domains.^38–40^ The protein variant with such an insert, borne by plasmid pBCL3-E55·FL (Table 1), was called TpiA^E55·FL^ (FL for *flexible linker*). In a second construct, an insert was made in the same site and the same length of that of TpiA^E55·FL^ but composed of the extended native PPV NIa site, i.e., the so-called NI_b_-CP junction.^32^ This consisted of the canonical target heptapeptide but added 5 additional naturally occurring amino acids at either side (i.e., DDGES·**NVVVHQA·**DERED). The resulting variant, called TpiA ^E55·NE^ (NE for *natural environment*), was encoded by plasmid pBCL3-E55·NE (Table 1).

As before, each of the new variants was passed through complementation assays to verify their activity *in vivo*, followed by inspection of their enzymatic performance *in vitro.* These showed the same outcome as before, i.e., the newly constructed TpiA variants were functional *in vivo* and *in vitro* (Figure S3). Once this was confirmed, an *in vivo* proteolytic assay was run by coexpressing each of these TpiA variants together with the NIa protease from the pPPV1 plasmid, followed by detection of the processed peptides by Western blot (Figure 5A). The results indicated that TpiA^E55·FL^ was hardly amenable to cleavage by NIa. In contrast, the TpiA^E55·NE^ variant underwent near-complete proteolytic degradation, which could be detected even at low NIa expression levels. Note that since the experiment was probed with anti-Etag antibodies, only the C-terminal fragment of TpiA was detected. The fragment identified here was 6 kDa lighter than the full length TpiA^E55·NE^, which is in good agreement with the expected removal of 55 amino acids from the N-terminal domain.

**Figure 5:**
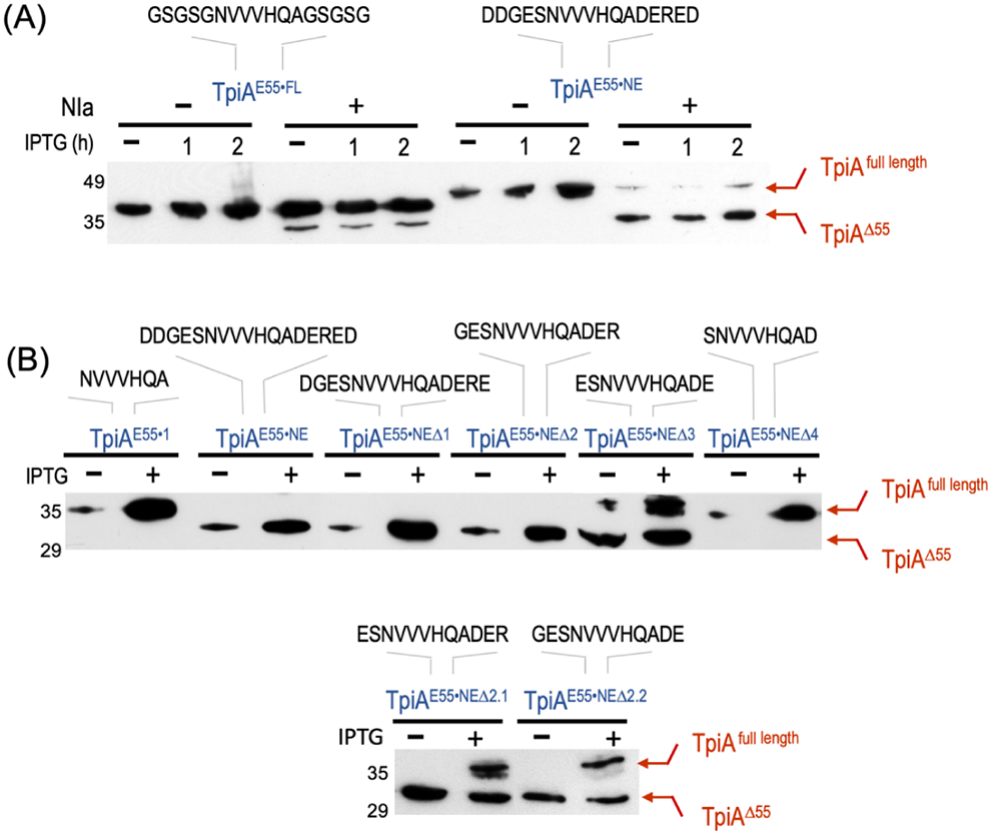
Optimization of the NIa protease recognition target. (A) Two types of extensions were introduced flanking the heptapeptide corresponding to the core NIa cleavage site to enhance the proteolytic performance of TpiA. First, five amino acids with a high propensity to form flexible loops were added on each side of the core sequence, leading to the TpiA^E55·FL^ variant (left). Second, the five flanking residues corresponded with the native sequence found adjacent to the heptapeptide in the PPV NIb–CP polyprotein, producing the TpiA^E55·NE^ variant. The specific sequences of each insert are indicated above each construct. (B) To further streamline the active E55·NE target site defined in panel A, additional TpiA variants with progressively shorter peptide extensions were generated, as indicated in the text and above the corresponding Western blot. In each case, the red arrows denote the corresponding full-length TpiA variant (TpiA ^full length^) and the faster-migrating proteolytic fragment lacking the N-terminal 55 residues (TpiA^Δ55^). The optimal NIa target site in TpiA was defined as the sequence GESNVVVHQADER, and thus, the TpiA^E55·NEΔ2^ variant was used in the following experiments.

These results clearly exposed that the lack of functionality of the core **NVVVHQA** as the target of the NIa protease in the structured context of the E55 site of TpiA was not due to an insufficient display of the target but to the need of an adequate amino acid context. This is in contrast with other known cases, where the same heptapeptide can be cleaved perfectly well when placed in nonstructured regions or hinge motifs in naturally occurring or engineered polyproteins.^26,41–44^

To further streamline an optimal NIa-cleavage site in the TpiA structure, we leveraged the data on TpiA^E55·NE^ to identify the minimal sequence of its 17 amino acid insertion that could still hold full amenability to proteolysis. To this end, we constructed and analyzed a collection of enzyme variants bearing serial amino acid deletions within the inserted NE peptide, such as removing 1, 2, 3, or 4 amino acids flanking the core heptapeptide. The removal of one or two of the most external amino acids of each side did not affect proteolysis efficiency, i.e., TpiA^E55·NEΔ1^ (pBCL3-E55·NEΔ1, insertion DGES·**NVVVHQA·**DERE) and TpiA^E55·NEΔ2^ (pBCL3-E55·NEΔ2, insertion GES·**NVVVHQA·**DER), respectively (Figure 5B). In contrast, the processing capability was reduced to half in the absence of the next flanking amino acid pair, i.e., the cleavage site flanked by 2 residues (pBCL3-E55·NEΔ3, TpiA^E55·NEΔ3^, insertion ES·**NVVVHQA·**DE) and was completely abolished in variant TpiA^E55·NEΔ4^ (plasmid pBCL3-E55·NEΔ4, insert S·**NVVVHQA·**D). Finally, to check whether either the G or R external residues of the TpiA^E55·NEΔ2^ insert could be removed while keeping their amenability to NIa proteolysis, we further constructed TpiA variants lacking one amino acid or the other, i.e., TpiA^E55·NEΔ2–1^ (pBCL3-E55·NEΔ2–1, ES·**NVVVHQA·**DER) and TpiA^E55·NEΔ2–2^ (pBCL3-E55·NEΔ2–2, GES·**NVVVHQA·**DE), respectively. Analysis of these two variants (Figure 5C) showed that both Gly and Arg seem to play a role in determining proteolytic competence in the structural context of TpiA since elimination of either reduced cleavage performance very significantly.

The outcome of these experiments was the identification of the peptide GES**NVVVHQA**DER inserted in E55 as the optimal NIa target site to bring about full and specific TpiA proteolysis. A structural prediction of the insert in the context of the TpiA protein is shown in Figure S4. Also, inspection of the same results indicated that expression of the NIa protease gene from the IPTG-inducible pPPV1 plasmid was leaky enough to lead to full cleavage of the target TpiA protein when containing the appropriate target site, an issue that—as addressed below—deserved attention for designing a fully TpiA-switchable strain.

### Engineering a Strain with a Fully Proteolizable Triosephosphate Isomerase

Once the TpiA variant most amenable to proteolysis by NIa was established, a specialized strain bearing a full genomic replacement of the native *tpiA* gene of *E. coli* W3110 by the equivalent *tpiA* ^*E55·NEΔ2*^ sequence was constructed, using an adaption of a genome editing method^45,46^ (Figure S5). Proteolytic processing of thereby edited and chromosomally encoded *tpiA* was tested in the presence of plasmid pPPV1, expressing the cognate protease (Figure 6A). Isogenic strain *E. coli* BCL3, harboring the *tpiA* gene with the C-terminal E-tag epitope but lacking the NIa recognition sequence (Table 1), was used as a negative wild-type-like control. Expression of *tpiA* was easily detected in either strain in Western blot experiments, at least until the late exponential phase, suggesting that the implantation of the protease-target peptide did not affect TpiA synthesis (Figure 6B, right panel). The band corresponding to the intact TpiA protein disappeared in the Western blot upon induction of protease expression, and a new band of lower weight, corresponding to the truncated TpiA protein, could be detected. This proved that the chromosomally borne TpiA protein engineered with the NIa target site was fully split by the cognate protease. These tests benchmarked the experimental system as they showed that the protease specifically recognized and split the entire pool of TpiA protein when inserted with an appropriate NIa recognition sequence. The plasmid-free, consolidated strain bearing a full genomic replacement of *tpiA* by an E-tagged *tpiA* ^*E55·NEΔ2*^ allele was named *E. coli* BCL4. Growth of this strain in minimal medium with glycerol was indistinguishable from that of the parental counterpart (Figure 6B, left panel). Consistent with these results, TpiA activities of the wild-type strain *E. coli* BCL3 and the isogenic strain *E. coli* BCL4 carrying *tpiA* ^*E55·NEΔ2*^ were indistinguishable in the absence of NIa. Induction of the cognate NIa protease resulted in a marked decrease in triosephosphate isomerase activity in *E. coli* BCL4, whereas the control strain *E. coli* BCL3 remained unaffected. These results indicated that protein cleavage destroys its functionality and demonstrated the biochemical performance of TpiA of *E. coli* BCL4 in a protease-dependent manner (Figure 6B right panel: compare blue and orange plots).

**Figure 6:**
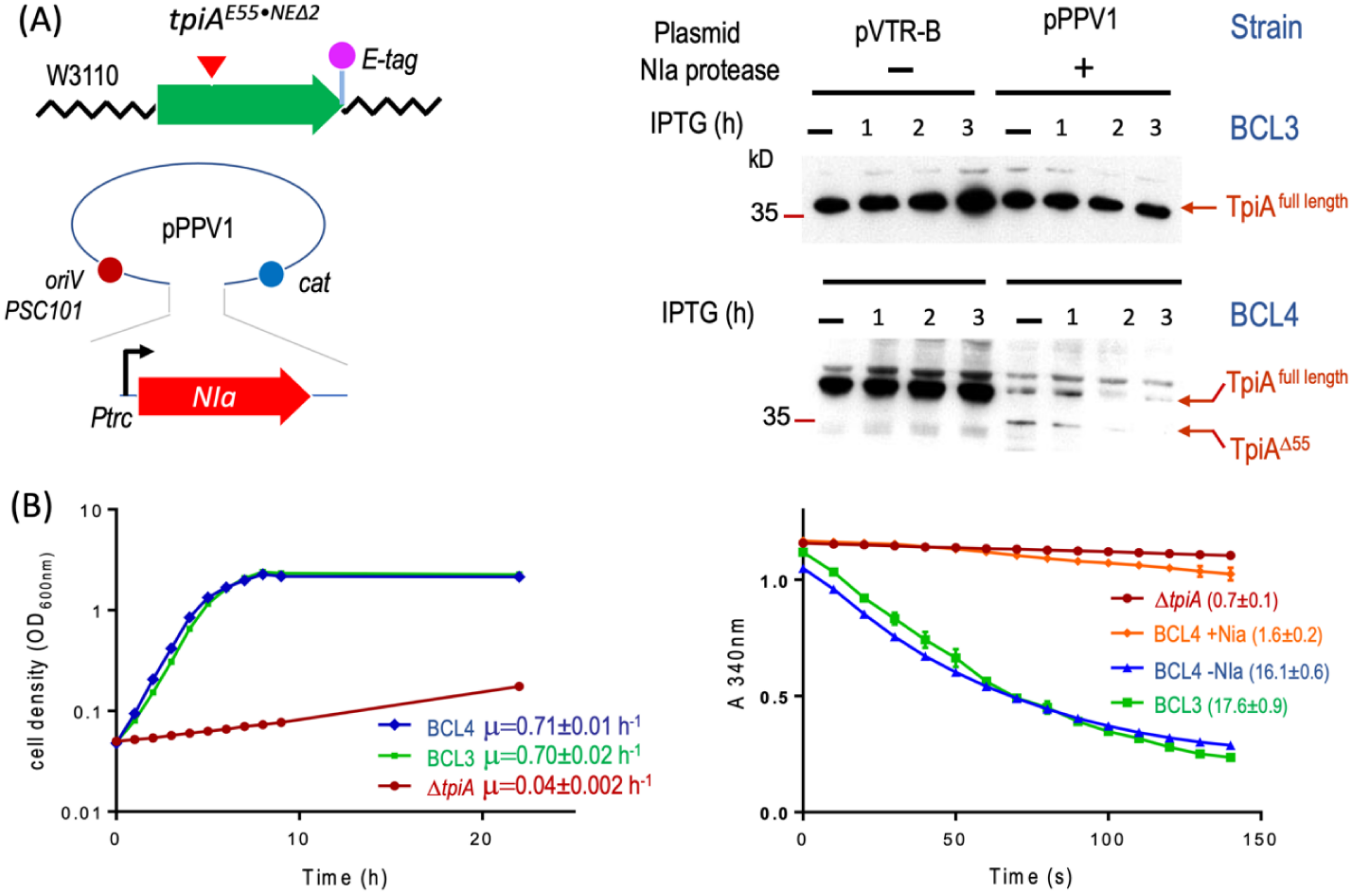
Performance of the *E. coli* BCL4 strain harboring the protease-sensitive TpiA^E55·NEΔ2^ variant. (A) Schematic representation of the experimental setup to test the performance of the chromosomal-borne TpiA^E55·NEΔ2^ mutant protein (strain BCL4) when transformed with plasmid pPPV1 driving the expression of the cognate protease from an IPTG inducible promoter (left panel), and analysis of proteolytic processing products along time after inducing the NIa-expression (right panel). Western blots were carried out with crude extracts obtained from cultures of both the NIa-sensitive BCL4 strain and the strain BCL3, harboring the tpiA-Etag gene that was used as a wild-type-like control, transformed with the NIa-expressing plasmid pPPV1 or the corresponding empty pVTR-B parental vector. Samples were taken before (time 0) and after one, two, or three h of induction. The full-length protein (slower mobility band, TpiA^full length^) or its C-terminal fragment devoid of the first 55 residues (faster mobility band, TpiA^Δ55^) indicated by red arrows were detected by anti-Etag antibodies. Molecular weight markers, run in parallel, are shown on the left side of the gels. (B) Growth of BCL4 strain in minimal medium supplemented with 1% glycerol as the sole carbon source, compared to the wild-type-like strain BCL3 and the TpiA-defective derivative, which were used as control strains. The specific growth rate of each strain is indicated (left panel). The right panel shows the enzymatic activity of soluble extracts obtained from strain BCL4 transformed with plasmid PPV1 under non-inducing conditions (blue line) or after 3 h of ITPG induction (orange line). The BCL3 strain was used as a positive control of activity, while the tpiA KO derivative was used as a negative control. Specific TpiA activities (mmol·mg^–1^·min^–1^) are indicated in brackets. Enzymatic experiments were done as explained in Figure 4 and in experimental procedures.

### Conditional Inactivation of TpiA Causes a Peak of Intracellular DHAP Accumulation

As discussed above, deletion of *tpi*A gene enables accumulation of DHAP from glucose or glycerol, but strongly impairs growth and ultimately activates methylglyoxal formation in *E. coli* ^12^ (Figure 1). This makes the mere genetic removal of *tpiA* a faulty strategy for the intracellular buildup of the triose. Instead, the strain *E. coli* BCL4 has the *tpiA* ^*E55·NEΔ2*^ gene that produces a conditionally active protein, and thus, it is expected to transiently accumulate DHAP when the isomerase activity is switched off through the action of the NIa protease. To test this hypothesis, *E. coli* BCL4 was transformed with the NIa-expressing plasmid pPPV1, and DHAP levels were quantified through LC-MS in both wild-type *E. coli* and the engineered strains with or without expression of the protease, using a *tpiA* defective strain as a control. In the absence of the protease, no significant amount of DHAP was detected in *E. coli* BCL4—as was the case for the wild-type parental strain (Figure 7). However, in the presence of the NIa protease, DHAP accumulation was detected in the strain carrying *tpiA* ^*E55·NEΔ2*^. Levels were similar to the full *tpiA*-knockout at relatively long growth times (i.e., cells in the early stationary phase). Therefore, DHAP buildup in cells with a transiently inactivated TpiA had a wild-type-like profile unless the expression of the NIa protease gene was induced. The experiments of Figure 7 also suggest that that DHAP can accumulate for a certain period without apparent diversion through MgsA. Yet, this issue was not entirely clarified, as the system for expression from a *P*_*trc*_ promoter of the NIa protease from plasmid pPPV1 is leaky, and there is always a degree of basal expression, particularly in multicopy vectors, that translates into a default decrease of the physiological levels of TpiA activity in cells bearing the plasmid, even without induction with IPTG. We therefore aimed to enhance the expression system to convert intracellular NIa production in *E. coli* BCL4 from an inducible mode to a strict ON/OFF control, enabling a true *hit-and-run* TpiA inactivation event.

**Figure 7:**
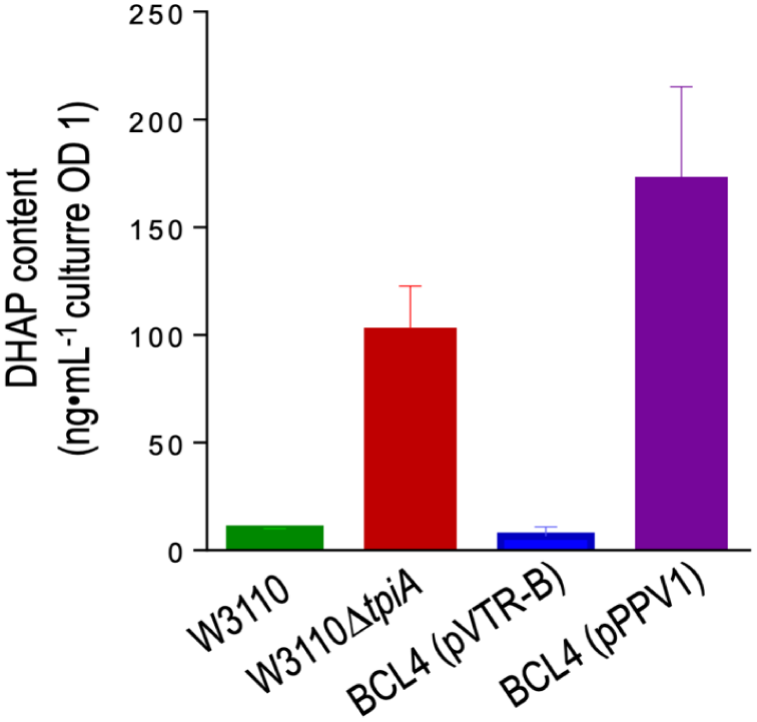
DHAP accumulation quantified by HPLC-MS. *E. coli* W3110 (green bar), the *tpiA* null strain (red bar), and the conditionally active *tpiA* strain BCL4 harboring plasmid pVTR-B (absence of NIa protease, blue bar) or with pPPV1 (carrying the NIa protease, purple bar) DHAP content was analyzed in bacteria grown to early stationary phase and is given as nanograms per milliliter of culture at an OD600 = 1. Data are the mean values of two independent experiments with two technical replicas each with the corresponding standard deviations.

### Synthetic Switch Enables Precise Spatiotemporal Removal of TpiA Activity

To submit TpiA activity to a rigorous expression switch, we passed the NIa gene sequence from plasmid pPPV1 to a vector with a digitalized version of the stringently controlled XylS-*Pm* regulatory device. Such a device, which has been described in detail before,^24^ results from implementation of a genetic circuit that combines transcriptional factors XylS and LacI and their cognate promoters with small RNAs that target translation initiation sequences of the gene of interest (GOI), thereby delivering high expression of such GOI only when cells are exposed to 3-methylbenzoate (3-*m*Bz). The plasmid construct bearing such a device with NIa is called pS238·NIa (Table 1), which affords absolute suppression of NIa expression in the absence of 3-*m*Bz and manifestation of the protease in its presence in a virtual ON/OFF fashion^24^ (Figure S6).

With plasmid pS238·NIa in hand, we transformed the NIa sensitive-*tpiA* ^*E55·NEΔ2*^ strain *E. coli* BCL4 and analyzed changes in DHAP and other related intracellular metabolites with rapid, full inactivation of the isomerase to inspect not only the efficacy of the switch but also the extent of the perturbation in the biochemical network of the cells. The resulting levels of such metabolites in the test strain with and without 3-*m*Bz induction were compared to those of the wild-type and *tpiA-*defective strains, which provided the controls for the experiment. As shown in Figure 8, the DHAP content of control *E. coli* BCL3 and the derivative bearing the proteolizable TpiA *E. coli* BCL4 without induction was kept at the same low level. But, as anticipated, DHAP levels in *E. coli* BCL4 increased steadily upon inactivation of TpiA through NIa protease induction of plasmid pS238·NIa, reaching 3-fold higher values after 1 h of 3-*m*Bz addition to the cultures.

**Figure 8:**
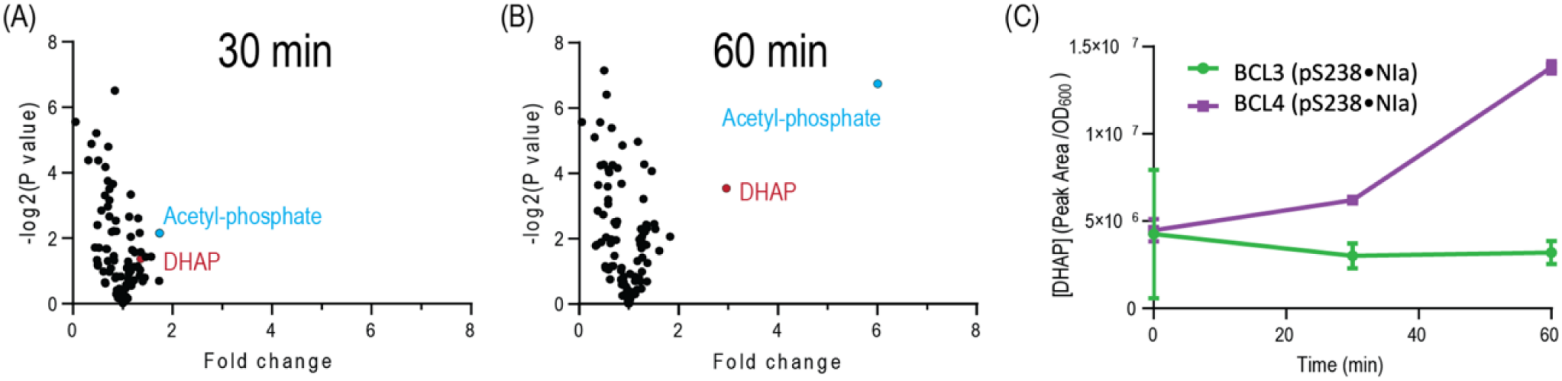
Metabolic changes imposed through conditional TpiA disruption. (A) Intracellular metabolite concentration changes after 30 min induction of the NIa protease from plasmid pS238·NIa with 1 mM 3mBz in *E. coli* BCL4. Note that none of the metabolites showed more than a 2-fold change compared to time 0 min. (B) After 60 min of induction, only DHAP and acetyl phosphate showed more than a 2-fold change. (C) Upon induction of *E. coli* BCL3 (bearing the noncleavable TpiA variant), DHAP levels did not show significant changes. In contrast, intracellular DHAP concentrations of *E. coli* BCL4 increased rapidly. Data shown are the mean values of two independent experiments, i.e., mean values ± standard deviation.

Broad inspection of more than 100 intracellular metabolites by nontargeted metabolomics (Data) allowed us to gain insight into the perturbations triggered by the sudden depletion of triosephosphate isomerase activity. To this end, strain *E. coli* BCL4 carrying pS238·NIa was grown in minimal medium with glucose. Metabolites were extracted right before induction with 1 mM 3-*m*Bz as well as 30 and 60 min after induction (Figure 8). The metabolism was largely undisturbed after 30 min of induction (Figure 8A). Given the rapid degradation of TpiA after 20 min of proteolytic processing, we expected to detect altered DHAP levels already after 30 min. Yet, DHAP levels were merely increased by 36% at that time (Figure 8). A closer inspection of the kinetic properties of TpiA reveals that the isomerase is very fast, with a *k*_cat_ > 9,000 s^–1^, indicating that TpiA is not a rate-limiting enzyme under physiological conditions^47–49^ Thus, the TpiA activity should be almost completely depleted before substantial accumulation of DHAP becomes evident. Additionally, a moderate increase in the level of DHAP is probably buffered by increased triglyceride synthesis, another sink of the target metabolite.

The metabolome landscape after 60 min of induction was rather different. At this point, almost the whole metabolic complement seemed rather unchanged, but DHAP levels showed a notable alteration (Figure 8B) that reached a threefold increase compared to the intracellular concentration prior to induction (Figure 8C). Such increased DHAP concentration was faster between 30 and 60 min compared to the first 30 min of proteolytic processing, which pinpoints a delayed onset of DHAP accumulation after induction. Interestingly, acetyl-phosphate, a low-molecular-weight, high-energy intermediate and secondary messenger molecule in *E. coli*,^50^ was quite high in protease-induced engineered cells. Acetyl-phosphate is part of the lower pyruvate metabolism via the phosphotransacetylase-acetate kinase (Pta-AckA) pathway. Although the physiological role of acetyl-phosphate is not fully understood, this intermediate is involved in overflow metabolism. In our system, rising acetyl-phosphate levels might indicate the onset of a metabolic response to the disruption of TpiA reaction (e.g., altered pyruvate or acetyl-coenzyme A levels, which could trigger overflow metabolism).

As control experiments, the same setup as above was conducted with wild-type *E. coli* strain W3110 and its Δ*tpiA* mutant derivative. While the wild-type strain had substantial metabolic changes following 60 min of induction, it did not accumulate any DHAP (Figure 8C and Data Metabolomics). Hence, the disturbance of metabolism in the wild-type strain was likely caused by its entrance into the late exponential phase during the 60 min of cultivation due to its higher growth rate. The Δ*tpiA* mutant showed, as expected, substantially increased DHAP levels, but this phenotype came at the expense of extremely crippled growth.

In summary, targeted elimination of TpiA activity driven by NIa-mediated cleavage of the TpiA protein leads to a protracted but rapid and consistent increase in DHAP production *in vivo* without much perturbation (at least during the time window of the experiment) of the remaining metabolic network. Our observations at the metabolome level (high DHAP levels both in the *ΔtpiA* strain and in the engineered strain with a proteolyzed enzyme) challenge the physiological importance of the methylglyoxal pathway for dealing with an excess of the triose.^16^ In any case, we advocate the TpiA hit-and-run strategy described above as a phenomenal tool to increase the intracellular production of DHAP in a fashion entirely controlled by an external user and regardless of the growth conditions of the strain prior to induction of the protease.

## Conclusion

The results of this work demonstrate the feasibility of achieving precise, temporally resolved modulation of a central metabolic function through targeted proteolysis. By introducing a cognate recognition sequence for the PPV NIa protease into a structurally permissive loop of *E. coli* TpiA and combining this modification with a digitalized regulatory module ensuring strict ON/OFF control of protease expression, we established a robust system for conditional post-translational inactivation of TpiA. The resulting *hit-and-run* proteolytic device enabled transient depletion of the TpiA activity and a corresponding rise in intracellular DHAP levels, while maintaining overall metabolic balance. This strategy circumvents the physiological drawbacks of permanent *tpiA* deletions, providing a controllable and reversible means of redirecting the glycolytic flux toward DHAP production.

Although this work basically reports a proof of concept of the technology, one can entertain the possibility that coupling DHAP accumulation to a downstream utilization module (e.g., aldolase-driven synthesis) would enable conversion of the accumulated intermediate into value-added products, thereby transforming the control strategy into an industrially relevant biosynthetic route. The hit-and-run proteolysis system could in this case function as a dynamic flux-gating module, temporally decoupling biomass formation from product synthesis and enabling precise control over precursor availability. Note that this approach can be generalized through the use of a transposon-based tool that directly enters the NIa protease recognition sequence without any need of structural information.^51,52^ In any case, we argue that the hereby described stratagem for engineering conditional phenotypes is a valuable addition to the toolbox of genetic devices for synthetic and systems biotechnology.

## Materials and Methods

### Strains, Plasmids, and Media

Strains and plasmids used in this work are listed in Table 1. Solid and liquid Luria–Bertani (LB) medium and M9 minimal medium (supplemented with suitable carbon sources) were amended, when required, with ampicillin (100 μg/mL), streptomycine (50 μg/mL), chloramphenicol (30 μg/mL), and isopropyl-D-1-thiogalactopyranoside (IPTG; 0.1 mM). *Escherichia coli* DH5α and XL1-Blue strains were used for standard cloning procedures, whereas *E. coli* CC118λpir was used as the host to propagate plasmids containing an R6K origin of replication.^53^ Wild-type *E. coli* W3110 was the host strain for *tpi*A genome modifications. Construction of plasmids carrying TpiA variants harboring NIa protease targets of different lengths within various sites, as described in the text and in Table 1, was made by inserting synthetic DNA segments within the *Pme*I site of plasmid pBCL3-E55, pBCL3-E160, or pBCL3-A195 (Table 1), encoding a TpiA protein carrying a pentapeptide insertion after the E55, E160, or A195 residue, respectively, obtained by linker scanning mutagenesis, as reported.^23^ DNA assemblies were produced from hybridized oligonucleotides tailored to codify the corresponding putative NIa recognition sequences described in this work. Mixtures of complementary oligonucleotides, listed in Table S1 (20 μM each in 250 mM Tris, pH 7.5), were heated in a water bath at 95 °C for 5 min, followed by slow cooling down overnight. Hybridized DNA samples were then ligated to plasmid pBCL3-E55, pBCL3-E160, or pBCL3-A195, digested with *Pme*I. Clones with only one insert in the right orientation were checked by PCR and further sequencing. The final construct containing a *tpi*A gene coding for an efficiently NIa-cleavable site (i.e., TpiA^E55·NEΔ2^, including the target site GESNVVVHQADER) was named pBCL3-E55·NEΔ2 for simplicity.^54^ Plasmid pACBSR was obtained from Scarab Genomics.^55^ All primers used (Table S1) were purchased from Sigma-Aldrich. Other gene cloning techniques and standard molecular biology procedures were carried out by using previously published protocols.^56^

### Cell Cultures, Protein Extracts, and TpiA Enzyme Assays

Cells transformed with specific plasmids were grown at 37 °C with vigorous shaking to an OD_600_ of 0.4–0.5 when expression of TpiA or both TpiA and NIa protease was induced by addition of IPTG (100 μM) for 3 h. Then, cells were harvested by centrifugation at 7,000 × g for 10 min at 4 °C. Cell pellets were disrupted by sonication in seven 15-s periods alternating with 1 min periods of cooling in ice. The crude cell lysate was centrifuged at 12,000 × g for 30 min at 4 °C to obtain soluble protein extracts suitable for enzymatic activity quantification. Protein concentration was measured by Bradford assay. The enzymatic activity of the triosephosphate isomerase in such extracts was monitored as the decrease of absorbance at 340 nm due to the oxidation of NADH in a coupled-enzyme assay based on the method of Plaut and Knowles,^57^ with modifications as described.^23^

### Protein Electrophoresis and Western Blot Analysis

Soluble cell extracts were separated by SDS-PAGE (10%) and electroblotted onto a PVDF Inmobilon-P membrane (Millipore). TpiA protein and its derivatives harbor an E-tag peptide fused at the C-terminus, which was employed for specific immunological detection in Western blot experiments. HRP-anti-E-tag conjugate antibodies (Pharmacia) were used at a final dilution of 1:5000. In this case, detection was carried out with a luminescence reaction using a solution of 1.25 mM luminol (Sigma-Aldrich), 40 mM luciferin (Roche), and 0.0075% (v/v) hydrogen peroxide (Sigma-Aldrich) in 100 mM Tris-HCl, pH 8. Alternatively, mouse-anti-E-tag antibodies (Amersham Biosciences, ref 27-9413-01) were used at a 1:10000 dilution in combination with antimouse antibodies (Sigma, ref. A2554) diluted 1:5000. In this case, the detection was performed with BM Chemiluminescence Blotting Substrate (POD; ref. 115694001), according to the supplier’s instructions (Merck).

### Chromosomal *tpiA* Gene Replacement

A suicide-plasmid-based method was used to modify *tpi*A gene in *E. coli* W3110 chromosome. A homology region of 530 bp length immediately downstream of the *tpi*A gene was amplified by means of polymerase chain reactions (PCRs) using 100 ng of genomic w3110 DNA as a template and oligonucleotides tpiA-down F and tpiA-down R (including a homing endonuclease site I-SceI at the end of the downstream homology region). Reactions were run by first setting an initial denaturalization for 4 min at 94 °C, followed by 35 cycles of denaturalization (1 min, 94 °C), annealing (1 min, 62 °C), extension (1 min at 75 °C), and final extension (7 min, 75 °C). The fragment obtained was digested with *Hind*III and ligated to the corresponding site of pBCL3–55-NIa. Ligation was transformed into the XL1 strain. Selection of a clone containing the insert in the right orientation was carried out by cleaving appropriate restriction sites located in and outside the inset. The construct was further checked by sequencing. Next, this plasmid was digested with *Not*I and the resulting 1450 bp fragment, containing the whole *tpiA* gene plus the downstream sequence, was isolated and ligated into plasmid pKNG101, producing the ISceI-sensitive suicide plasmid pKNG101-tpiA, which was transformed in strain *E. coli* CC118λpir for plasmid maintenance. The suicide plasmid carrying the mutant *tpiA* allele was next transformed into the target strain *E. coli* W3110 and plated in LB supplemented with Sm. Resulting cointegrates were then pooled and transformed with the helper plasmid pACBSR, expressing the homing endonuclease I-*Sce*I. Cells were plated in LB amended with Sm and *Cm* to verify the presence of both plasmids. A colony from this transformation was inoculated in LB supplemented with chloramphenicol and 0.2% l-arabinose to induce I-*Sce*I production and incubated for 7 h at 37 °C. Appropriate dilutions of this culture were plated in LB or LB supplemented with *Cm*, Sm, or both antibiotics, to verify cointegration resolution performance. Sm-sensitive colonies were tested by PCR to verify the structure of the resulting *tpiA* allele upon cointegrate resolution. All possible outcomes, i.e., strains containing the *tpiA* gene inserted only with the NIa site, only with the Etag site (strain *E. coli* BCL3) or with both (strain BCL4), were obtained. The strain BCL4 was used for further experiments together with BCL3, which was used as a wild type-like control (as it is the noncleavable parental version of the gene).

### Quantification of DHAP

For dihydroxyacetone phosphate (DHAP) quantification, cells were grown in mineral minimum medium M9 with 0.2% glucose containing the appropriate antibiotic and inducer at 37 °C. The biomass of 8 mL of each of the samples, taken at the late exponential-early stationary phase of growth (OD_600_ = 0.8–1.1), was collected by fast centrifugation (14000 rpm, 20 s) and the pellets were immediately frozen in liquid N_2_ to quench the cell metabolome.^58^ DHAP was extracted from cells by treating the biomass with 25 mM ammonium acetate buffer (pH 7.2) in 60% ethanol at 70 °C for 1 min, as described previously.^59^ The residues from the dried samples were resuspended in 100 μL of Milli-Q water. DHAP analysis was performed on a Varian 1200L Triple Quadrupole MS equipped with 2 Varian ProStar model 210 HPLC pumps and a Gemini analytical column, C_18_, 110A, (150 mm × 4.6 mm, 5 μm, Phenomenex) with 5 μm packing (Phenomenex). The mass spectrometer was operated in negative ionization electrospray mode (ESI). The mobile phases consisted of acetonitrile (CH_3_CN, mobile phase A) and Milli Q water containing 0.1% formic acid (mobile phase B). Chromatography was performed at 25 °C with a flow rate of 0.2 mL/min. The gradient started with 5% A, increased linearly to 95% A over 10 min, and kept at that proportion for 15 min. Finally, re-equilibration was made with 5% A for 10 more min. The injection volume was 10 μL, and the retention time for DHAP was 12 min. Quantification was performed by comparison with a five-point calibration curve using external standards (0.05–5 ng/μL) and nonweighted linear regression. In all cases, each bar represents the mean value of the corresponding parameter ± the standard deviation of two independent experiments.

### Analysis of Intracellular Metabolites

Single colonies of the strains were grown overnight in 10 mL of M9 medium supplemented with 20 mM glucose and kanamycin at 37 °C . These overnight cultures were used to inoculate 50 mL of the same medium in 250 mL shaken flasks to an initial optical density (OD_600_) of 0.05 and grown at constant shaking at 37 °C. Periodically, samples were withdrawn to determine the optical density of the culture. Upon reaching an optical density of 0.5, the cultures were induced with 1 mM 3-methylbenzoate. With the addition of the inducer, as well as 30 and 60 min after initiating induction, samples were withdrawn for determination of intracellular metabolite levels. To this end, 2 mL of culture were vacuum-filtrated (0.45 μm PVDF, 47 mm, Durapore), and the biomass was quenched by immediately inverting the filter into a Petri dish with 1 mL of a water:methanol:acetonitrile (20:40:40% v/v) solution at −20 °C. All liquid was transferred to a reaction tube, and the filter was extracted again with 1 mL of quenching solution. Both extracts were merged in the reaction tube and centrifuged for 10 min at 13,000 g to remove insoluble particles. Subsequently, the supernatant was transferred to a new reaction tube and tried at 30 °C at reduced pressure (Concentrator Plus, Eppendorf, Hamburg, Germany). The samples were stored at −80 °C. Prior to analysis, the sediment was reconstituted in 100 μL of deionized water, and insoluble particles were removed by centrifugation at 17,000 × g for 10 min. Metabolites were quantified using LC-MS/MS according to the method of McCloskey et al.,^60^ and the chromatograms were analyzed using the MultiQuant software^61^ (Sciex, CA, USA).

## Supplementary Material





## Notes

PCR primers used in this work (Table S1); relevant sequences of the plasmids containing TpiA variants used in this work (Table S2); control assay to test NIa protease activity encoded in plasmid pPPV1, analyzed by means of Western blot of cell extracts harboring the plasmids indicated above each panel (Figure S1); performance of TpiA^E160·1^ and TpiA^A195·1^ protein variants (Figure S2); functional assessment of TpiA variants with extended insertions: TpiA^E55·NE^ and TpiA^E55·FL^ (Figure S3); AlphaFold prediction of the structure of the TpiA^E55·NED2^ protein variant (Figure S4).; overview of the procedure used to perform *tpiA* allelic exchange (Figure S5); comparison of the proteolytic performance of the NIa protease expressed from two expression systems­(Figure S6) (PDF)
Intracellular concentrations of major metabolites in different strains under induced or noninduced protease condition (Data) (XLSX)
