# Supplementary material for: Precision Proteolysis of Triosephosphate Isomerase of Escherichia coli Boosts Dihydroxyacetone Phosphate Biosynthesis
Source: ACS Synth Biol. 2026 Mar 3;15(3):1166–77. doi: 10.1021/acssynbio.5c00870 (PMC13010800; doi:10.1021/acssynbio.5c00870)
Supplement: Supplementary file 1 [file sb5c00870_si_001.pdf]

Supporting Information to Calles et al.

Supporting Table S1. PCR Primers used in this work

Oligonucleotide	Sequence
NIa-FO1	P-G***AACGTGGTGGTGCATCAGGCG***AA
NIa-RO1	P-TT***CGCCTGATGCACCACCACGTT***C
NIa-Flex F	P-GGGTAGCGGCAGCGGC***AACGTGGTGGTGCATCAGGCG***GGTAGCGG CAGCGGCAA
NIa-Flex R	P-TTGCCGCTGCCGCTACC***CGCCTGATGCACCACCACGTT***GCCGCTGC CGCTACCC
NIa-EN F	P-GGATGATGGTGAAAGC***AACGTGGTGGTGCATCAGGCG***GATGAACGCG AAGATAA
NIa-EN R	P-TTATCTTCGCGTTCATC***CGCCTGATGCACCACCACGTT***GCTTTCACCAT CATCC
NIa-EN1 F	P- GGATGGTGAAAGC***AACGTGGTGGTGCATCAGGCG***GATGAACGCGAAAA
NIa-EN1 R	P-TTTTCGCGTTCATC***CGCCTGATGCACCACCACGTT***GCTTTCACCATCC
NIa-EN2 F	P-GGGTGAAAGC***AACGTGGTGGTGCATCAGGCG***GATGAACGCAA
NIa-EN2 R	P-TTGCGTTCATC***CGCCTGATGCACCACCACGTT***GCTTTCACCC
NIa-EN3 F	P-GGAAAGC***AACGTGGTGGTGCATCAGGCG***GATGAAAA
NIa-EN3 R	P-TTTTCATC***CGCCTGATGCACCACCACGTT***GCTTTCC
NIa-EN4 F	P-GAGC***AACGTGGTGGTGCATCAGGCG***GATAA
NIa EN4 R	P-TTATC***CGCCTGATGCACCACCACGTT***GCTC
NIa-EN 2.1F	P-GGAAAGC***AACGTGGTGGTGCATCAGGCG***GATGAACGCAA
NIa-EN 2.1R	P-TTGCGTTCATC***CGCCTGATGCACCACCACGTT***GCTTTCC
NIa-EN 2.2F	P-GGGTGAAAGC***AACGTGGTGGTGCATCAGGCG***GATGAAAA
NIa-EN 2.2R	P-TTTTCATC***CGCCTGATGCACCACCACGTT***GCTTTCACCC
tpiA-down F	CGACGGAAGCTTGTCTGACAGGTGCCGGATTTC
tpiA-down R	CCGTCGAAGCTT**ATTACCCTGTTATCCCTA**CTTGGCAGGCGCGTGATTTTA TG

Restriction sites used for cloning purposes are underlined. The homing endonuclease site ISceI is denoted in bold-underlined characters. Bold italics indicate sequences coding for aminoacids of the putative NIa recognition site.

Supporting Table S2. Relevant sequences of the plasmids containing TpiA variants used in this work.

**Insertion plasmids constructed in this work**	**Length and point of insertion in *tpi*A gene**	**Insertion Sequence**
pBCL3-E55	Pentapeptide insertion after E55 residue in *tpiA* gene	E(55)-GCLNK-G(56)
pBCL3-E160	Pentapeptide insertion after E160 residue in *tpiA* gene	E(160)-GCLNK-G(161)
pBCL3-A195	Pentapeptide insertion after A195 residue in *tpiA* gene	A(195)-KVFKH-K(196)
pBCL3-E55•1	Thirteen aa long insertion, including NIa core site, after E55 residue in *tpiA* gene	E(55)-GCL**NVVVHQA**KNK-G(56)
pBCL3-E160•1	Thirteen aa long insertion, including NIa core site, after E160 residue in *tpiA* gene	E(160)-CL**NVVVHQA**KNIE-G(161)
pBCL3-A195•1	Twelve aa long insertion, including NIa core site, after E55 residue in *tpiA* gene	A(195)-KVF**NVVVHQA**KH-K(196)
pBCL3-E55•NE	Twenty-three aa long insertion composed of the NIa core cleavage site plus five flanking residues in their native arrangement, inserted after residue E55 in *tpiA* gene	E(55)-GCL**DDGESNVVVHQADERED**KNK-G(56)
pBCL3-E55•FL	Twenty-three aa long insertion consisting of the NIa core site flanked by five Gly/Ser residues on each side, positioned after residue E55 in the *tpiA* gene.	E(55)-GCL**GSGSGNVVVHQAGSGSG**KNK-G(56)
pBCL3-E55•ENΔ1	Twenty-one aa long insertion composed of the NIa core cleavage site plus four flanking residues in their native arrangement, inserted after residue E55 in *tpiA* gene	E(55)-GCL**DGESNVVVHQADERE**KNK-G(56)
pBCL3-E55•ENΔ2	Nineteen aa long insertion composed of the NIa core cleavage site plus three flanking residues in their native arrangement, inserted after residue E55 in *tpiA* gene	E(55)-GCL**GESNVVVHQADER**KNK-G(56)

pBCL3-E55•ENΔ3	Seventeen aa long insertion composed of the NIa core cleavage site plus two flanking residues in their native arrangement, inserted after residue E55 in *tpiA* gene	E(55)-GCL**ESNVVVHQADE**KNK-G(56)
pBCL3-E55•ENΔ4	Fifteen aa long insertion composed of the NIa core cleavage site plus one flanking residues in their native arrangement, inserted after residue E55 in *tpiA* gene	E(55)-GCL**SNVVVHQAD**KNK-G(56)
pBCL3-E55•ENΔ2−1	Eighteen aa long insertion composed of two flanking residues before and three after the NIa core cleavage site, in their native arrangement, inserted after residue E55 in *tpiA* gene	E(55)-GCL**ESNVVVHQADER**KNK-G(56)
pBCL3-E55•ENΔ2−2	Eighteen aa long insertion composed of three flanking residues before and two after the NIa core cleavage site, in their native arrangement, inserted after residue E55 in *tpiA* gene	E(55)-GCL**GESNVVVHQADE**KNK-G(56)

The pentapeptide generated through a previous linker-scanning mutagenesis experiment, is underlined in each case while the internally specific sequence introduced within them are highlighted in bold.

Supporting Figure S1. Control assay to test NIa protease activity encoded in plasmid pPPV1 analyzed by means of Western blot of cell extracts harboring the plasmids indicated above each panel.

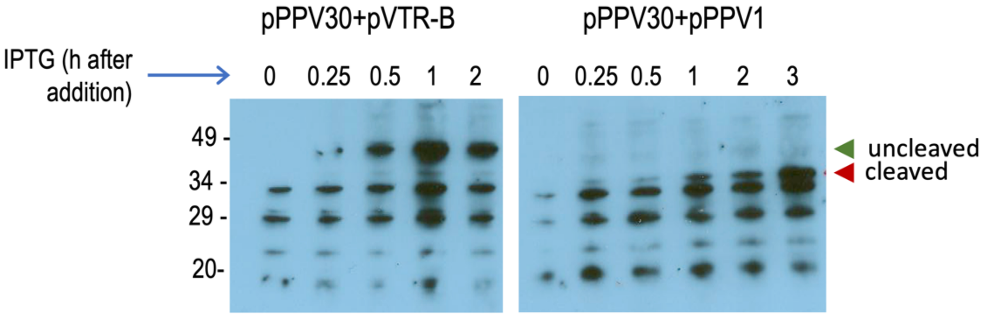

Plasmid pPPV30 contains a protease-less cDNA fragment spanning nt 8514-9319 of PPV, cloned in a pUC19 plasmid [1], while *pPPV1* carries a 0.6 kb fragment from pPPVS20, encoding the protease domain of the NIa protein from the plum pox potyvirus [2], placed in pVTR-B plasmid [3]. Expression of both plasmids is under the control of *E. coli* borne IPTG-inducible promoters (*Plac* in pPPV30 and *Ptrc* promoter in pPPV1). Non-processed polypeptide, of approximately 40 kDa, was detected in cells containing pPPV30 plus pVTR-B control plasmid, which do not contain the protease cistron (see left panel) while the activity of the protease encoded in pPPV1 was enough to cleave the excess wild-type NI_b-CP site (see right panel), as shown by the disappearance of the intact polypeptide encoded by pPPV30 (approximately 40 KDa, green arrow) and the presence of processed peptides with faster mobility (36.4 kDa, red arrow). The anti-PPV CP serum used for immunological detection of PPV polypeptides was a generous gift of Juan Antonio Garcia´s Laboratory.

Supporting Figure S2. Performance of TpiA$^{E160\bullet1}$ and TpiA$^{A195\bullet1}$ protein variants.

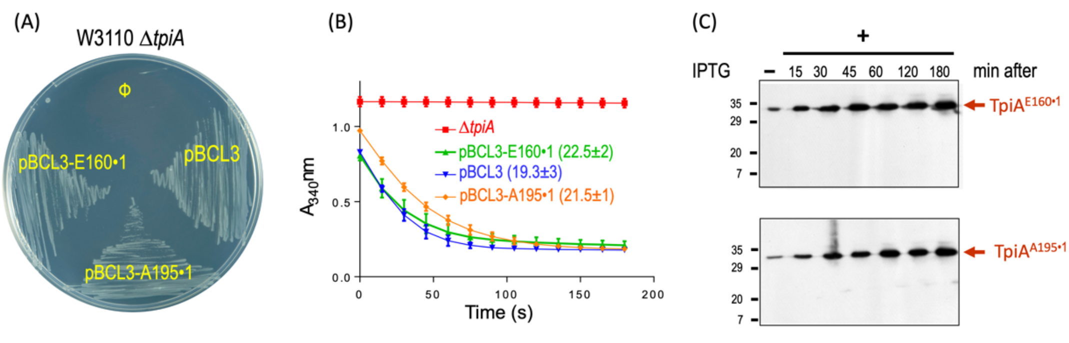

Proteins were expressed from plasmids pBCL3-E160•1 and pBCL-A195•1, respectively and evaluated in terms of (A) complementation of a tpiA-deficient strain (B) Enzymatic specific activities (mmol·mg^{-1}·min^{-1} shown in brackets) and (C) proteolytic susceptibility to the cognate NIa protease. As explained in the text, both TpiA variants fully complemented and showed wild type-like activity but were not cleaved by a NIa protease produced *in trans*.

Supporting Figure S3: Functional assessment of TpiA variants with extended insertions: TpiA$^{E55\bullet NE}$ and TpiA$^{E55\bullet FL}$

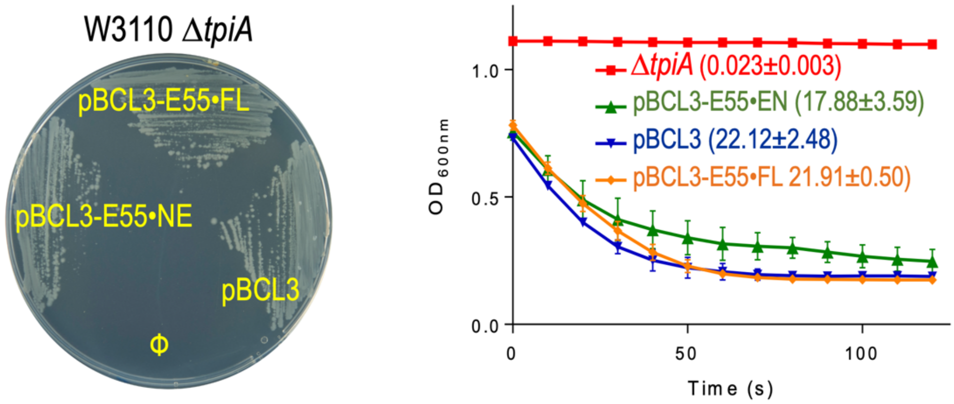

TpiA variants containing the NIa core target sequence, either flanked by flexible linker residues (TpiA$^{E55\bullet FL}$) or by the native amino acids from PPV1 (TpiA$^{E55\bullet NE}$), were expressed from plasmids pBCL3-E55•FL and pBCL3-E55•NE, respectively. The empty plasmid (Φ) was used as negative control. Their functionality was first assessed *in vivo* by complementation of a *tpiA*-deficient strain (left panel) and *in vitro* by measuring enzymatic activity (right panel), prior to evaluating susceptibility to NIa-mediated cleavage, as shown in Fig. 5.

Supporting Figure S4. AlphaFold prediction of the structure of the TpiA$^{E55•NE\Delta2}$ protein variant, with the Etag epitope fused to the C-terminal portion of the enzyme, which is predicted to continue alfa helix number 13, having the last portion as an unstructured region.

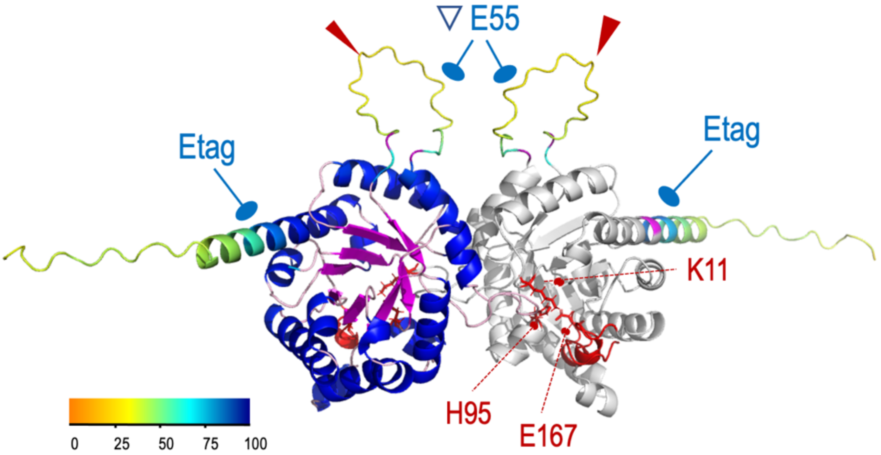

The 23 aa long peptide, harboring the cognate cleaving site of NIa protease (GCLDDGESNVVVHQ↓ADEREDINK), was placed after the permissive site in E55 residue, as described [4] and is predicted to form an unstructured coil motif, preceding the L2 loop, which comprises G58 to I61 residues. Amino acids marked in magenta stand for the starting and end point of the inserted peptide while the red arrow indicates the cleaving site of NIa (between Q and A in the protease target sequence). The active center of the enzyme is colored in red and it is composed by and active lid (residue 165 to 175), including the key E167 residue, which together with K11 and H95 amino acids conform the crucial catalytic triad. One of the monomers of TpiA has been colored according to secondary structure elements (helices in blue color, beta sheets in magenta and loops in light pink color) while the other is grey colored to highlight the active site and the modifications made to the enzyme, i.e. the internal E55 insertion and the C-terminal Etag epitope. Residues acids added up to the TpiaA protein, i.e. the C-terminal Etag and the NIa target sequence, are colored on a PLDDT basis as shown in the corresponding color bar scale

Supporting Figure S5. Overview of the procedure used to perform *tpiA* allelic exchange.

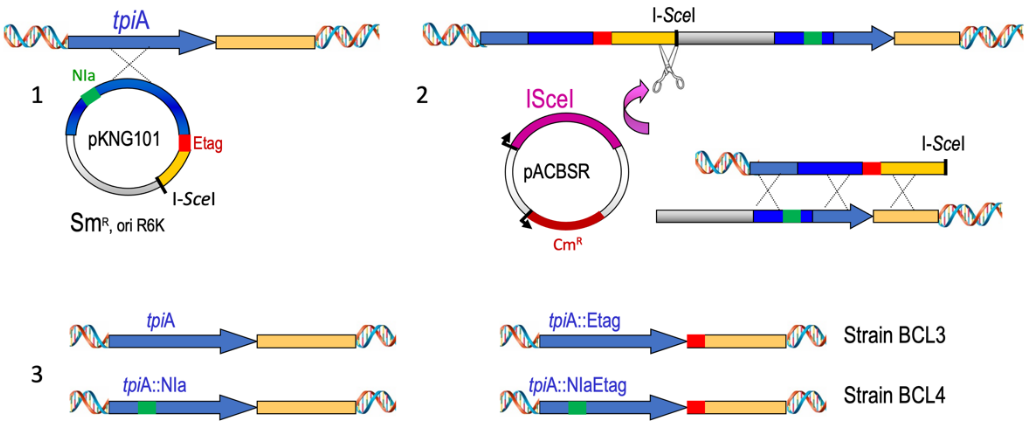

The suicide plasmid pKNG101, carrying the *tpiA*$^{E55•NED2}$ allele together with a downstream homology region ending in a unique I-SceI restriction site, was introduced into *E. coli* W3110 to generate cointegrates (Step 1). These cointegrates were subsequently resolved by inducing expression of the I-SceI endonuclease from plasmid pACBSR (Step 2). Resolution events yielded either wild-type revertants or one of three possible recombinant alleles, depending on the crossover point. Strain BCL4 carries the *tpiA* allele encoding both the C-terminal E-tag and the optimized NIa target sequence positioned after residue E55, whereas strain BCL3 retains only the E-tag epitope. A third recombinant strain containing only the NIa target sequence, but lacking the C-terminal E-tag, was also recovered. A detailed step-by-step description of the procedure is provided in the main text. Suicide plasmid pKNG101 harboring the TpiA$^{E55•NED2}$ variant plus a downstream homology ending in an unique I-SceI endonuclease site was transformed in strain W3110 to generate cointegrates (1) that were thereafter resolved by expression of the ISceI endonuclease from plasmid pACBSR [5-6]. Cointegrate resolution lead to revertants or to three different allele replacements, depending on the crossover point. Thus, strain BCL4 carried a tpiA mutant allele with both the C-terminal E-tag sequence and the NIa target sequence defined as optimal, placed after E55 residue while the strain BCL3 included only the E-tag epitope. A third strain, containing only the NIa target site but lacking the C-terminal E-tag sequence, was also obtained as result of this procedure.

Supporting Figure S6. Comparison of the proteolytic performance of the NIa protease expressed from two expression systems. Redrawn from [7].

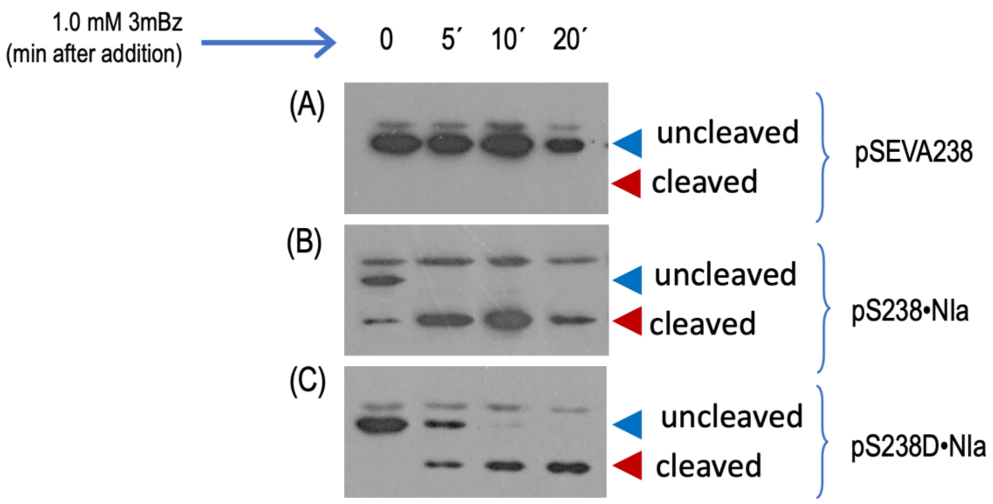

(A) Insert-less vector. (B) Standard XylS-Pm expression system (pSEVA238•NIa). (C) digitalized version pS238D•NIa.

REFERENCES IN Supporting Information

(1) Garcia, J. A., Riechmann, J. L., Martín, M. T., and Laín, S. (1989) Proteolytic activity of the plum pox potyvirus NIa-protein on excess of natural and artificial substrates in *Escherichia coli. FEBS Letters 257*, 269-273.

(2) García, J. A., Riechmann, J., and Lain, S. (1989) Proteolytic activity of the plum pox potyvirus NIa-like protein in *Escherichia coli. Virology 170*, 362-369.

(3) Pérez-Martin, J., and de Lorenzo, V. (1996) VTR expression cassettes for engineering conditional phenotypes in *Pseudomonas*: activity of the *Pu* promoter of the TOL plasmid under limiting concentrations of the XylR activator protein. *Gene 172*, 81-86.

(4) Calles, B., Pitarch, B., and de Lorenzo, V. (2025) The Structural Permissiveness of Triosephosphate Isomerase (TpiA) of *Escherichia coli*. *ChemBioChem 26*, e202400863.

(5) Pósfai, G., Kolisnychenko, V., Bereczki, Z., and Blattner, F. R. (1999) Markerless gene replacement in *Escherichia coli* stimulated by a double-strand break in the chromosome. *Nucl Acids Res 27*, 4409-4415.

(6) Kolisnychenko, V., Plunkett, G., Herring, C. D., Fehér, T., Pósfai, J., Blattner, F. R., and Pósfai, G. (2002) Engineering a reduced *Escherichia coli genome. Genome Research 12*, 640-647.

(7) Calles, B., Goñi-Moreno, Á., and de Lorenzo, V. (2019) Digitalizing heterologous gene expression in Gram-negative bacteria with a portable ON/OFF module. *Mol Syst Biol 15*, e8777.
